# Nitrogen Addition Altered the Effect of Belowground C Allocation on Soil Respiration in a Subtropical Forest

**DOI:** 10.1371/journal.pone.0155881

**Published:** 2016-05-23

**Authors:** Tongxin He, Qingkui Wang, Silong Wang, Fangyue Zhang

**Affiliations:** 1 Institute of Applied Ecology, Chinese Academy of Sciences, Shenyang, China; 2 University of Chinese Academy of Sciences, Beijing, China; 3 Huitong National Research Station of Forest Ecosystem, Huitong, China; Tennessee State University, UNITED STATES

## Abstract

The availabilities of carbon (C) and nitrogen (N) in soil play an important role in soil carbon dioxide (CO_2_) emission. However, the variation in the soil respiration (R_s_) and response of microbial community to the combined changes in belowground C and N inputs in forest ecosystems are not yet fully understood. Stem girdling and N addition were performed in this study to evaluate the effects of C supply and N availability on R_s_ and soil microbial community in a subtropical forest. The trees were girdled on 1 July 2012. R_s_ was monitored from July 2012 to November 2013, and soil microbial community composition was also examined by phospholipid fatty acids (PLFAs) 1 year after girdling. Results showed that R_s_ decreased by 40.5% with girdling alone, but N addition only did not change R_s_. Interestingly, R_s_ decreased by 62.7% under the girdling with N addition treatment. The reducing effect of girdling and N addition on R_s_ differed between dormant and growing seasons. Girdling alone reduced R_s_ by 33.9% in the dormant season and 54.8% in the growing season compared with the control. By contrast, girdling with N addition decreased R_s_ by 59.5% in the dormant season and 65.4% in the growing season. Girdling and N addition significantly decreased the total and bacterial PLFAs. Moreover, the effect of N addition was greater than girdling. Both girdling and N addition treatments separated the microbial groups on the basis of the first principal component through principal component analysis compared with control. This indicated that girdling and N addition changed the soil microbial community composition. However, the effect of girdling with N addition treatment separated the microbial groups on the basis of the second principal component compared to N addition treatment, which suggested N addition altered the effect of girdling on soil microbial community composition. These results suggest that the increase in soil N availability by N deposition alters the effect of belowground C allocation on the decomposition of soil organic matter by altering the composition of the soil microbial community.

## Introduction

Soil respiration (R_s_) is the primary pathway for carbon dioxide (CO_2_) emission from the soil to the atmosphere and is estimated to be 80–100 Pg C·yr^−1^ [1]. The C released from the soil through R_s_ is equivalent to 10-fold C emission from fossil fuel combustion. Therefore, a slight change in the R_s_ rate will significantly influence the atmospheric CO_2_ concentration [2]. Previous studies suggested that photosynthate allocation in the roots and soil nitrogen (N) availability significantly affected R_s_ [3–5]. The amount of photosynthate-derived C allocated to the soil through the roots was found to change under CO_2_ elevation and N deposition [6–8]. N deposition also altered soil N availability, and consequently could affect R_s_ [9, 10]. However, the response of R_s_ to the combined changes in belowground C allocation and N availability in forest ecosystems remains incompletely understood.

The removal of the bark and phloem in stem girdling can terminate the allocation of photosynthates from the tree crown to the roots [3, 4]. This process has been widely used to separate soil heterotrophic respiration (R_H_) from autotrophic respiration (R_A_) because of its minimal disturbance to the soil–root–microbe system [3, 4, 11, 12]. Girdling can also be used to detect the effect of belowground C allocation on R_s_ [11–13]. Girdling generally reduces R_s_ [14], but the magnitude of reduction varies among different forest ecosystems. For instance, girdling in a Norway spruce forest and boreal pine forest resulted in a reduction in R_s_ by approximately 50% [3, 14], whereas R_s_ was only reduced by 14%–24% in a eucalyptus plantation [4, 13]. These studies suggest that the influence of girdling on R_s_ is system specific because of the distinct rooting systems and soil properties among different forests [3, 4]. Therefore, the effect of girdling on R_s_ across a wide range of forest ecosystems should be investigated.

Soil N availability is another important factor controlling R_s_ [9, 10, 15]. Several studies have found that N addition or fertilization decreased R_s_ in forest ecosystems [16–18]. However, in other some studies, N addition showed no effect on R_s_ [19, 20], or even increased it [21, 22]. These contradicting results may result from the different responses of R_H_ and R_A_ to N addition [10]. R_H_ generally exhibits a similar negative response to N addition, especially in forest ecosystems [23]. However, the effects of N addition on R_A_ were inconsistent, showing positive [24, 25] or negative results [26], which induced different responses of R_s_ to N addition. Previous studies also indicated that the positive response of R_s_ to N addition was mainly attributed to the increase of R_A_ [24, 27]. The mean N deposition in China increased from 13.2 kg N ha^−1^·yr^−1^ in the 1980s to 21.1 kg N ha^−1^·yr^−1^ in the 2000s [28]. This increase may enchance the soil N availability and alter the response of R_s_ to belowground C allocation. The increase in N availability usually improves the gross primary productivity and net primary productivity by stimulating forest growth and biomass production [29]. However, some studies indicated that high N deposition reduced the belowground C allocation or the investment by plants in fine roots [23], resulting in a direct negative effect on R_s_ [30]. Therefore, a better understanding of the soil C cycle under global change is essential to investigate the combined effects of C supply and N addition on R_s_.

Soil microbial community is tightly coupled with R_s_ [15, 31–33]. Soil microbes are considered C-limited, whereas plant productivity is frequently N-limited [34]. Photosynthate C allocated to soil is a key C source for microbes and influences soil microbial community and activities [35–37]. Some studies have investigated the effects of reducing C supply on soil microbes among different forest ecosystems [20, 36, 38]. Soil N availability also affects soil microbial biomass, activities and community composition by altering belowground C allocation or litter quality [31], thereby affecting soil microbial respiration. A meta-analysis suggested that the reduction in soil microbial biomass or a shift in microbial community composition was responsible for the changes in R_s_ induced by N addition in forest ecosystems [23]. However, the effects of the combined changes of C supply and N addition on R_s_ through soil microbial community remain incompletely understood.

In this study, photosynthate C allocation and soil N availability were manipulated by stem girdling and N addition to evaluate the effects of C supply and N availability on R_s_ in the global change context in a subtropical forest in Southern China. Girdling reduces the contribution of R_A_ to R_s_, and on the basis of the reduction in C supply after girdling, R_s_ would be decreased. Moreover, girdling also affects R_H_ by altering the soil microbial community composition. Based on the abovementioned, we hypothesized that girdling would reduce R_s_ rapidly. Increased soil N availability decreases the soil organic C to be decomposed by microbes [18]. Therefore, we also hypothesized that N addition would decrease R_s_ and enhance the effects of girdling on R_s_ and microbial community composition.

## Materials and Methods

### Site description

This study was conducted at the Huitong National Research Station of Forest Ecosystem (26°40′–27°09′N and 109°26′–110°08′E) in the Hunan Province, subtropical China. This region has a typical mid-subtropical monsoon climate with a mean annual temperature of 16.5°C and mean annual precipitation of 1200 mm. The soil derived from the Sinian Period gray-green slate was classified as ultisol according to the USDA soil taxonomy.

This study was conducted in a *Cunninghamia lanceolata* forest that was established in 1986. The stand density was 2,500 trees ha^−1^ at planting and approximately 1,200 trees ha^−1^ when this study began. No fertilizer or lime was added after planting. The primary understory vegetation included *Rubus rosifolius*, *Pteridium aquilinum*, *Maesa japonica*, *Parathelypteris chinensi*s, and *Microlepia marginata*. *C*. *lanceolata* has shallow roots, which were mainly distributed in the 0–40 cm soil layer.

### Experimental design

Six plots (6 m × 6 m) were set up in the *C*. *lanceolata* plantation in June 2012. The plots were 30–40 m apart, and each plot contained about five trees. The trees in three of the six plots were girdled on 1 July 2012, whereas the remaining three plots served as controls. The trees were girdled by removing the bark and cambium in a 10 cm band around the circumference of the trunk at breast height. The understory in the six plots was mowed monthly. Each plot was divided into two subplots in early November 2012 to investigate the combined effects of N addition and girdling on the soil processes. 2% NH_4_NO_3_ solution was added once a year in the below subplot at a rate of 100 kg N ha^−1^·yr^−1^. The same amount of water was added to the control plots. This experiment included four treatment groups, namely, control plots (CT), plots with N addition (N), plots with girdling (G), and plots with girdling and N addition (GN). The amount and pattern of litterfall were not changed by girdling. The leaves began to wither 17 months after girdling, and trees continued to sprout from the stems. The sprouts below the girdling area were removed.

### Soil respiration

Four polyvinyl chloride collars with an inner diameter of 10.4 cm and a height of 8 cm, were permanently installed in the middle of each subplot. The soil collars were pushed 5 cm into the mineral soil to seal the R_s_ chamber. R_s_ was measured using a Li-cor 8100 infrared gas analyzer (Li-cor Inc. Lincoln, NE, USA). Soil temperature and volumetric moisture were simultaneously measured. R_s_ was monitored twice in the first month after girdling, and then monthly thereafter. R_s_ was measured thrice for each collar and the R_s_ used was the average of the three measurements.

### Soil sampling and analysis

Mineral soils samples (0–10 cm) were collected on 4 July 2013 after the litter layer was removed. Eight soil cores (diameter = 25 mm) were obtained from each subplot at even distances between two neighboring girdled trees, and then mixed to a composite soil sample. Soil samples were immediately taken to the laboratory and sieved using a 2 mm mesh. Roots and stones were manually removed. A portion of the soil samples was stored at 3°C to determine soil microbial biomass C (MBC), dissolved organic C (DOC), and inorganic N (NH_4_^+^-N and NO_3_^−^-N), or was freeze-dried to analyze soil microbial community. The other portions were air-dried in the laboratory to determine soil organic carbon (SOC) and total nitrogen (TN).

Soil MBC was determined using the fumigation extraction method and was calculated using the following equation [39]: MBC = *K*_EC_ × 2.2, where *K*_EC_ is the C extracted from the fumigated soil minus the C extracted from the non-fumigated soil. The non-fumigated C content is referred to hereafter as K_2_SO_4_-extractable C, which is a proxy for soil DOC [35]. The inorganic N was analyzed by extracting 10 g of fresh soil samples with 2 M KCl in a 1:4 soil-to-solution ratio for 1 h. NH_4_^+^-N concentration in the solution was measured with indophenol blue and NO_3_^−^N concentration was determined by copperized cadmium reduction method coupled with modified Griess-Ilosvay method using a UV-160 spectrophotometer, at 625 nm and 210 nm respectively [40]. SOC and TN were determined using an automated C/N analyzer (Vario MAX CN, Elementar Co. Hanau, Germany). Soil pH was determined using a pH meter with a 1:2.5 (w: v) mixture of soil and KCl solution. The soil microbial community was assayed using phospholipid fatty acids (PLFAs) [5]. The fatty acids used as biomarkers for specific groups of soil organisms are listed in Table 1.

**Table 1:** Fatty acids used in the analysis of microbial community composition in the study.

Soil microbial groups	Diagnostic fatty acids	Reference
**Bacteria**	i14:0; i15:0; a15:0; i16:0; 16:1ω7t; i17:0; a17:0; 18:1ω7c; cy19:0	[41]
**Fungi**	18:1ω9; 18:2ω6,9	[42]
**Gram-positive bacteria**	i14:0; i15:0; a15:0; i16:0; i17:0; a17:0	[42–44]
**Gram-negative bacteria**	16:1ω7t; 17:1ω8c; 18:1ω7c; cy17:0; cy19:0	[43]

The prefixes “a” and “i” indicate antiso- and iso-branching, respectively, and “cy” indicates a cyclopropane fatty acid.

### Calculation and statistical analysis

We calculated the R_H_ and R_A_ as follows:

$$R_{H}=R_{G};R_{A}=R_{CT}-R_{G}$$

Where R_G_ is the respiration rate measured from G plots, and R_CT_ is the respiration rate measured from CT plots.

The main and interactive effects of girdling and N addition on R_s_, soil temperature, and moisture were investigated using repeated measures of two-way ANOVA. The effects of girdling and N addition on the soil properties and microbial community were tested using two-way ANOVA. Repeated measures of one-way ANOVA was used to detect the effect of N addition on R_H_ and R_A_. Significant differences between the various treatments were tested with Tukey HSD test. Regression analysis was performed to evaluate the influences of soil temperature and soil moisture on R_s_. Principal component analysis (PCA) was used to separate and group the samples based on their soil microbial groups, as represented by specific PLFA biomarkers. PCA and ANOVA were performed using SPSS17.0 software (SPSS, Chicago, IL, USA).

## Results

### Soil environmental conditions

**Fig 1:**
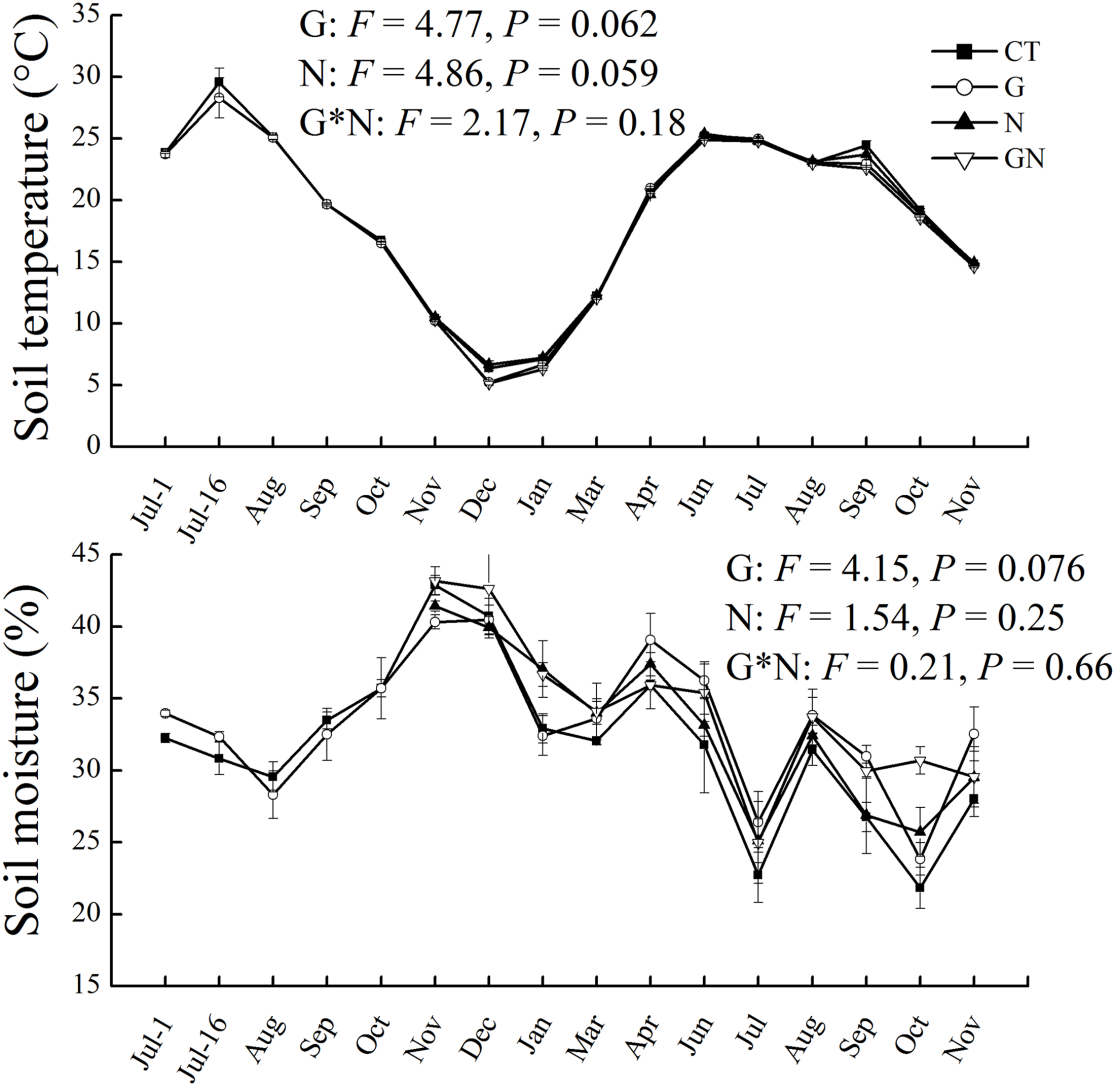
Mean monthly dynamics of soil temperature (5 cm) and volumetric moisture (0–5 cm) from July 2012 to November 2013 under different treatments. Error bars indicate standard error. CT, control; G, girdling; N, N addition; GN, girdling and N addition. The repeated measures of two-way ANOVA was used to analyze the main and interactive effects of girdling and N addition on soil temperature and moisture.

Soil temperature and moisture did not differ among treatments (Fig 1). Girdling (7.8 ± 0.8 mg kg^-1^) significantly enhanced soil NO_3_^−^-N by 202% compared with CT (2.6 ± 0.3 mg kg^-1^) (Table 2). N addition (5.6 ± 0.5 mg kg^-1^) also significantly increased soil NO_3_^−^-N compared with CT. GN significantly increased soil NH_4_^+^-N (22.6 ± 1.7 mg kg^-1^) and NO_3_^−^-N (9.6 ± 1.3 mg kg^-1^) by 16% (*P* < 0.05) and 269% (*P* < 0.01) compared with CT (NH_4_^+^-N, 19.5 ± 0.8 mg kg^-1^) respectively. The SOC, TN contents, and pH were unchanged by girdling or N addition.

**Table 2:** Results of two-way ANOVA for soil properties as dependent on girdling and N addition, and their interactions. The F-ratios are presented, together with their level of significance.

	df	SOC (g kg^-1^)	TN (g kg^-1^)	DOC (mg kg^-1^)	MBC (mg kg^-1^)	NH_4_^+^ -N (mg kg^-1^)	NO_3_^-^ -N (mg kg^-1^)	pH (KCl)
**G**	1	1.46	1.53	0.55	0.76	1.97	14.3**	0.11
**N**	1	0.13	0.02	0.78	2.42	0.52	5.02*	0.95
G*N	1	1.2	1.53	3.21	2.46	2.91	0.15	2.65

*P < 0.05.

**P < 0.01.

G, girdling; N, N addition.

### Soil microbial community composition

Girdling and N addition significantly altered soil microbial biomass, and the effect of N addition was greater than the effect of girdling (Table 3). Girdling significantly decreased total PLFAs (35.6 ± 1.9 nmol·g^-1^), bacteria (16.5 ± 1.3 nmol·g^-1^) and Gram-negative bacteria (8.1 ± 0.5 nmol·g^-1^) by 16.0%, 20.3% and 22.0% respectively, compared with CT (42.4 ± 3.2, 20.7 ± 1.1 and 10.4 ± 1.0 nmol·g^-1^ respectively). N addition reduced the total PLFAs (31.1 ± 0.3 nmol·g^-1^), bacteria (12.7 ± 0.2 nmol·g^-1^), Gram-positive bacteria (7.5 ± 0.3 nmol·g^-1^) and Gram-negative bacteria (5.2 ± 0.4 nmol·g^-1^) by 26.7%, 38.6%, 27.2% and 50.0% respectively, and increased the ratio of Gram-positive: Gram-negative by 43.1% compared with CT. Girdling with N addition had a greater effect on soil microbial community composition than girdling or N addition alone, reducing the total PLFAs, bacteria, Gram-negative, and Gram-positive bacteria compared with CT (*P* < 0.05).

**Table 3:** Results of two-way ANOVA for concentrations of PLFAs and two PLFA ratios as dependent on girdling and N addition, and their interactions. The F-ratios are presented, together with their level of significance.

	df	Total PFLAs	Bacteria	Fungi	Fungi:bacteria	G+	G-	G+:G-
**G**	1	5.85*	5.47*	0.89	0.04	2.57	5.17*	0.29
**N**	1	20.5**	30.8**	4.24	2.64	8.07*	40.3**	7.6*
G*N	1	0.6	2.72	0.02	0.57	0.74	3.48	0.6

G- and G+ indicate Gram-negative bacteria and Gram-positive bacteria, respectively.

*P < 0.05.

**P < 0.01.

**Fig 2:**
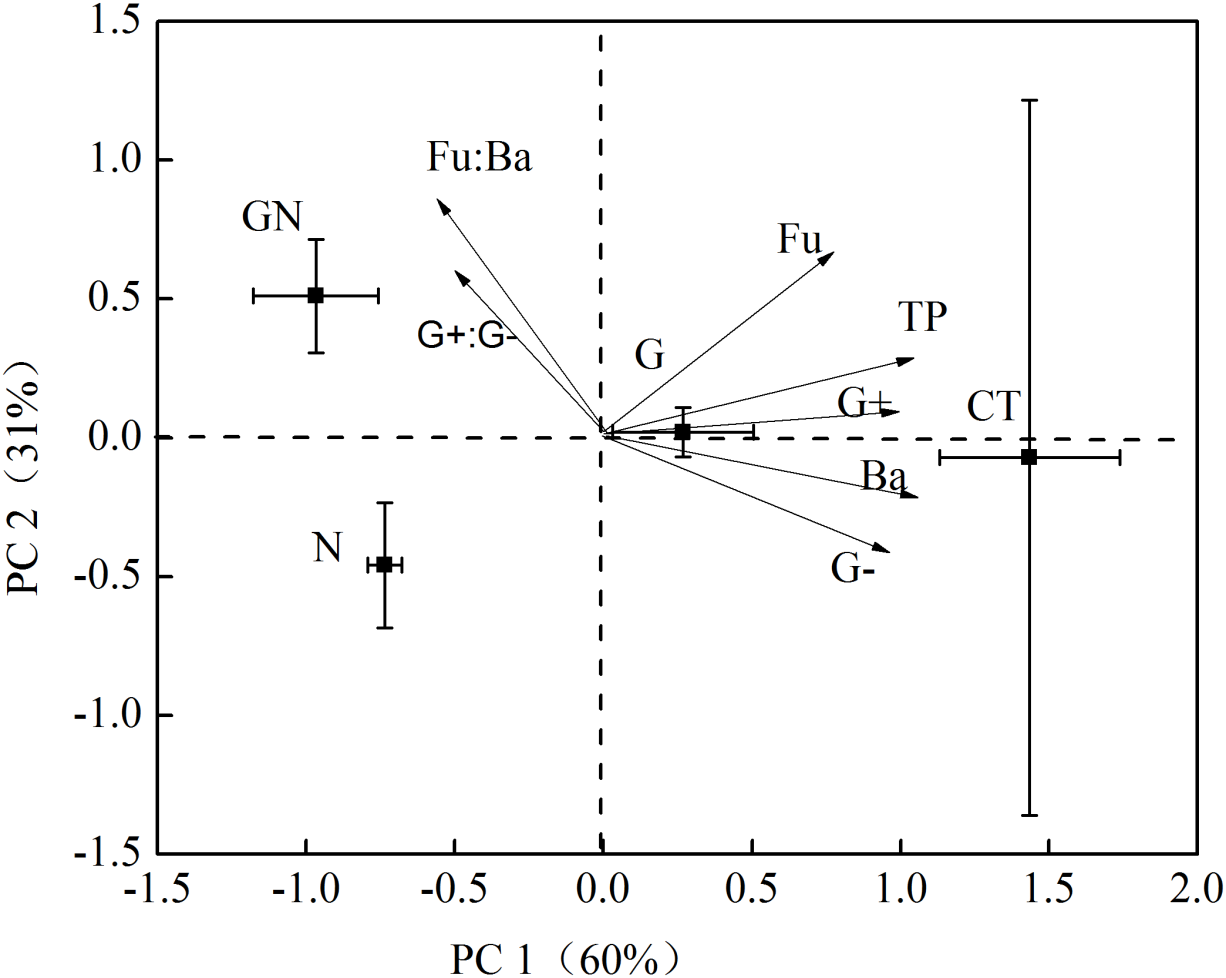
Principal component analysis (PCA) of PLFA data from soil under all treatments. (*n* = 3). Whiskers show standard error of the mean of plot score values; solid squares show the PCA weighted loading values of microorganisms. PLFA biomarker abbreviations: TP, total PLFAs; G+, gram-positive bacteria; G−, gram-negative bacteria; Ba, bacteria, Fu, fungi. CT, control; G, girdling; N, N addition; GN, girdling and N addition.

The PCA analysis based on soil microbial groups as indicated by PLFA biomarkers clearly separated the control and treatment plots (Fig 2). The first principal component (PC1), which accounted for 60% of the total variation, mainly reflected the influence of girdling, N addition, and their interaction on soil microbial community. These vectors illustrated that girdling increased the ratio of fungi:bacteria and G+:G- in N addition plots strongly discriminated communities on the second principal component (PC2), which accounted for 31% of the variation (Fig 2).

### Soil respiration

**Fig 3:**
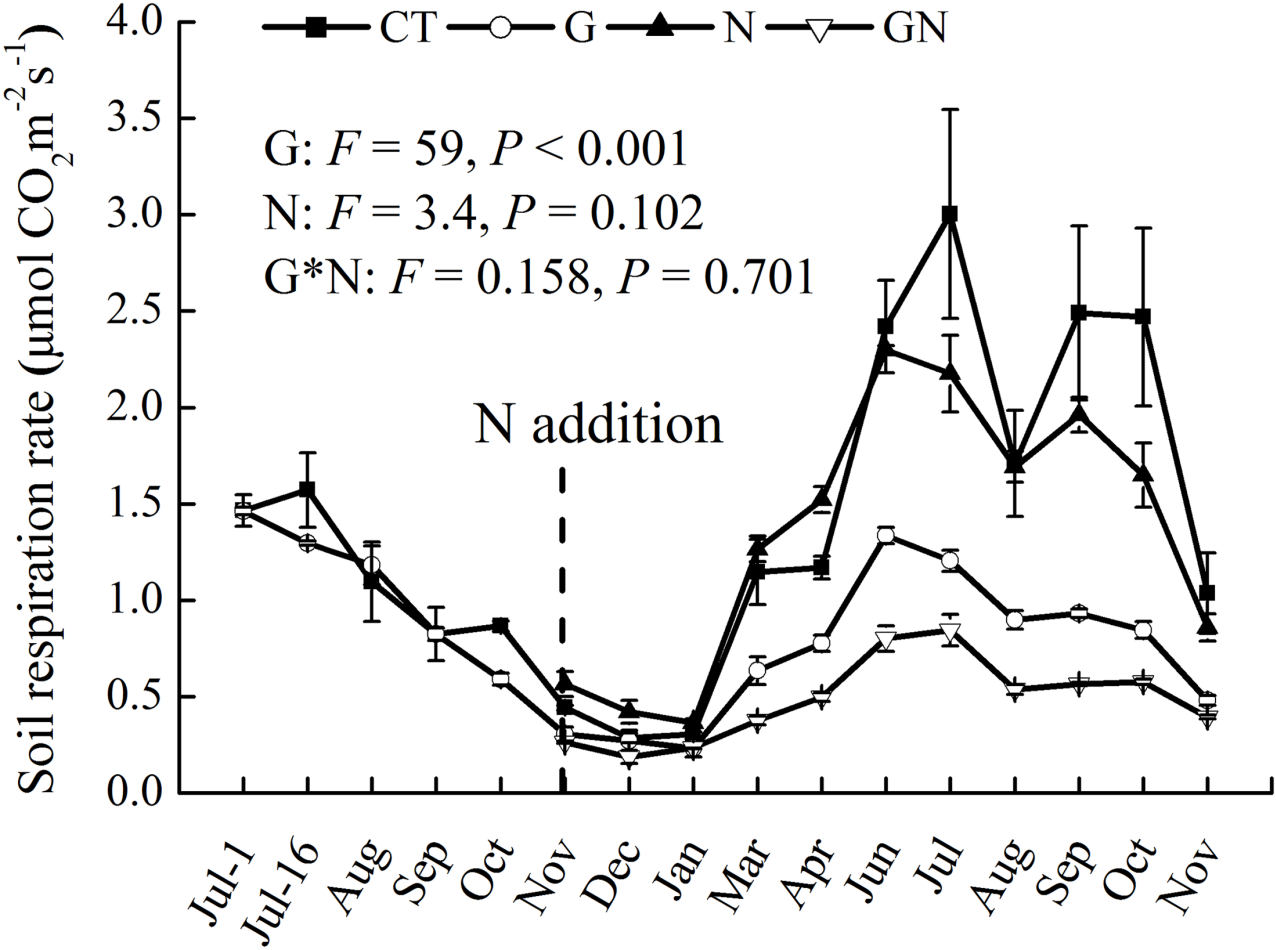
Mean monthly dynamics of soil respiration from July 2012 to November 2013 under different treatments. Error bars indicate standard error (*n* = 3). CT, control; G, girdling; N, N addition; GN, girdling and N addition. The repeated measures of two-way ANOVA was used to analyze the main and interactive effects of girdling and N addition on soil respiration.

**Fig 4:**
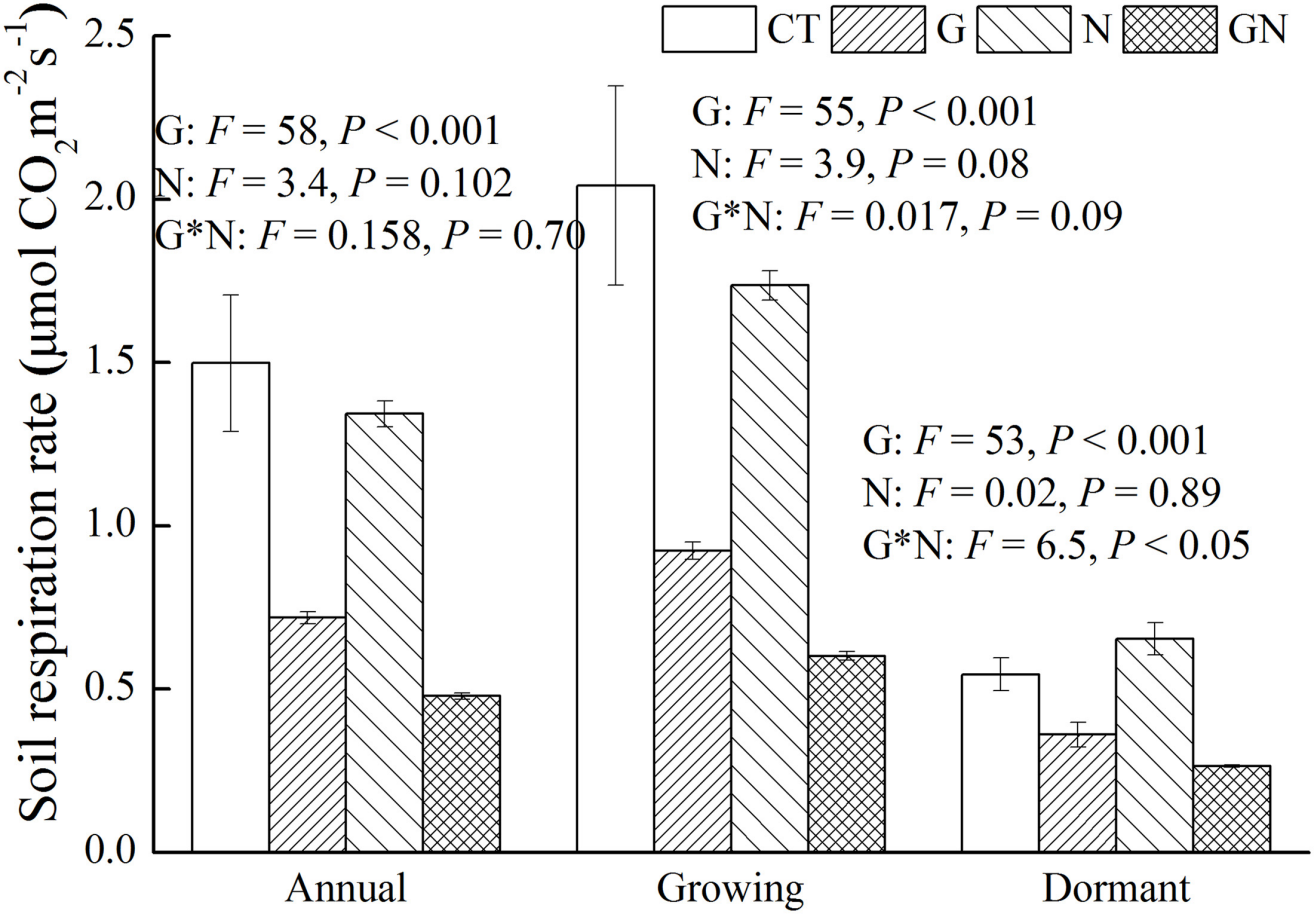
Mean soil respiration in annual, growing and dormant season under different treatments. Error bars indicate standard error (*n* = 3). CT, control; G, girdling; N, N addition; GN, girdling and N addition. The repeated measures of two-way ANOVA was used to analyze the main and interactive effects of girdling and N addition on soil respiration across different seasons.

**Fig 5:**
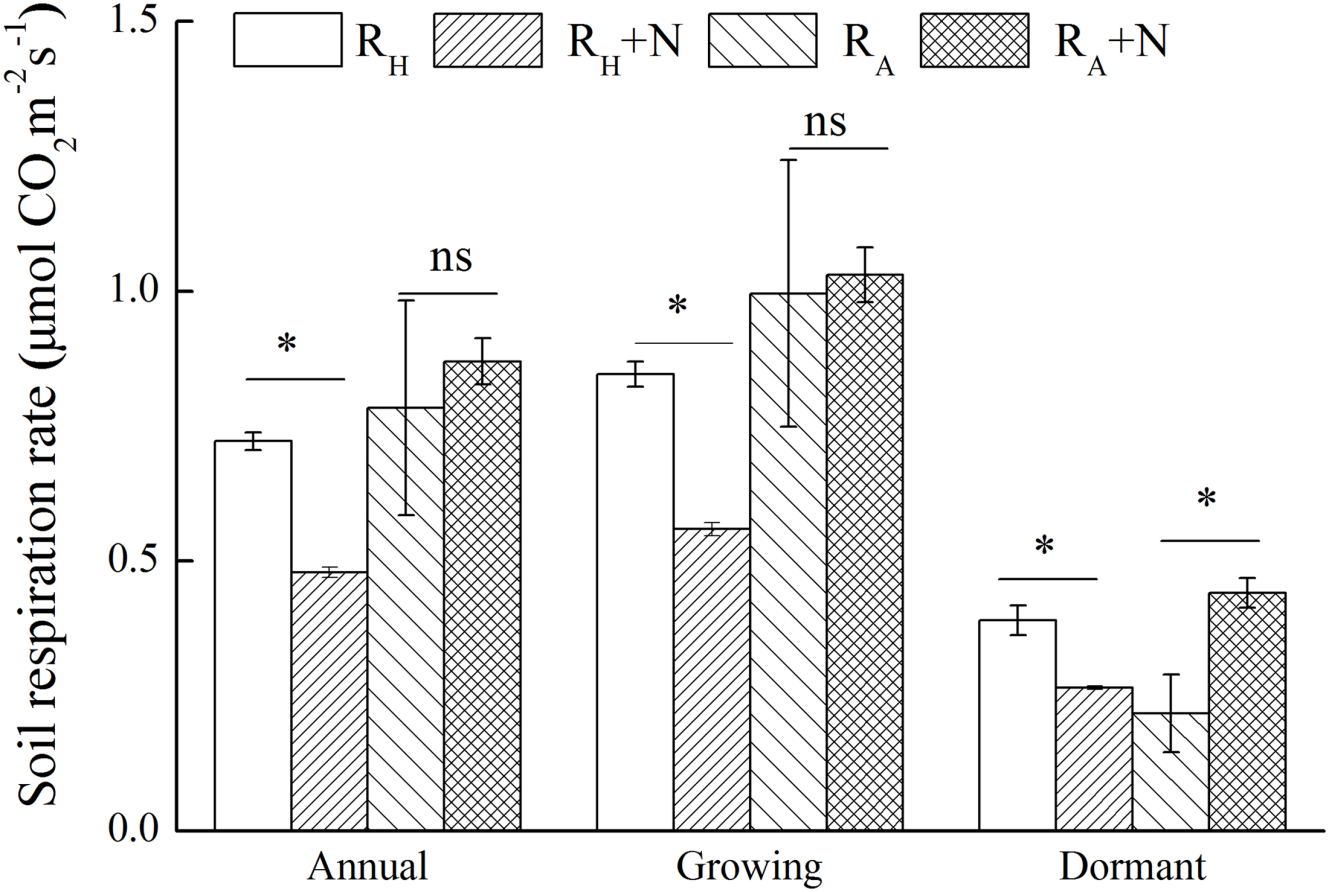
Mean soil heterotrophic (R_H_) and autotrophic respiration (R_A_) under control and N addition treatments in annual, growing and dormant season. Error bars indicate standard error (*n* = 3). The asterisks on the error bars denote significant effect of N addition based on repeated measures of one-way ANOVA across different seasons.

R_s_ showed strong seasonal patterns under different treatments. R_s_ was lower during the dormant season (December–March) than during the growing season (April–November) (Fig 3). R_s_ did not decrease in the first three months after girdling (Fig 3) but significantly decreased by 40.5% during the experimental periods after girdling compared with CT (Fig 4). N addition did not alter total R_s_. However, a significant effect of the interaction between N addition and girdling on R_s_ was found during the dormant season. Moreover, girdling with N addition significantly decreased annual R_s_ by 62.7% compared with N addition plot, which was higher than the decrease in R_s_ induced by girdling alone. The reduction in R_s_ ranged from 33.9% in G to 59.5% in GN during the dormant season, and from 54.8% in G to 65.4% in GN during the growing season compared with CT (Fig 4). N addition significantly decreased R_H_ by 33.3% and 32.0% during the growing and dormant seasons, respectively, but significantly increased R_A_ by 102% during the dormant season (Fig 5).

**Fig 6:**
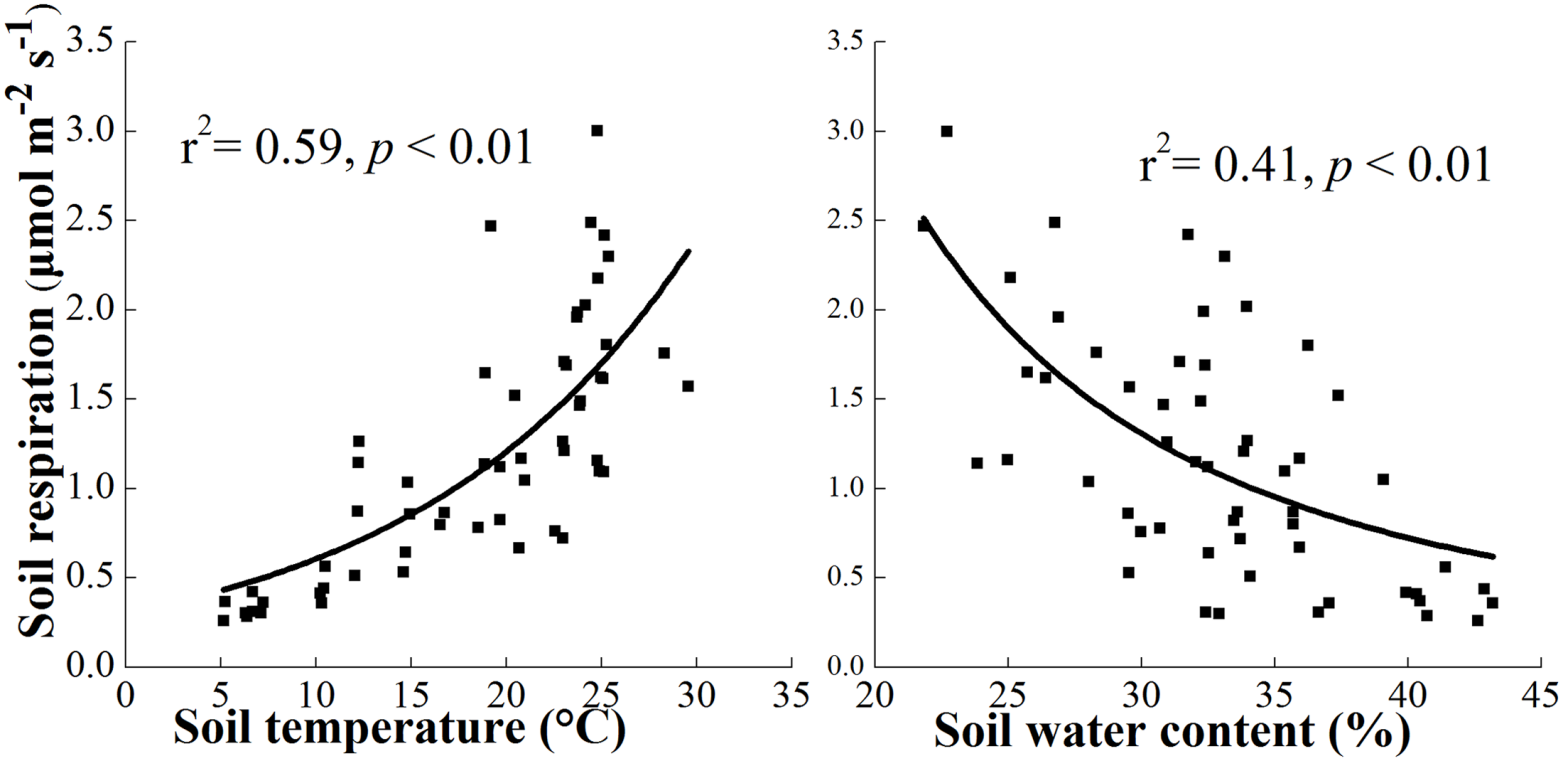
Relationships between soil respiration and soil temperature (5 cm) and volumetric moisture (0–5 cm).

**Fig 7:**
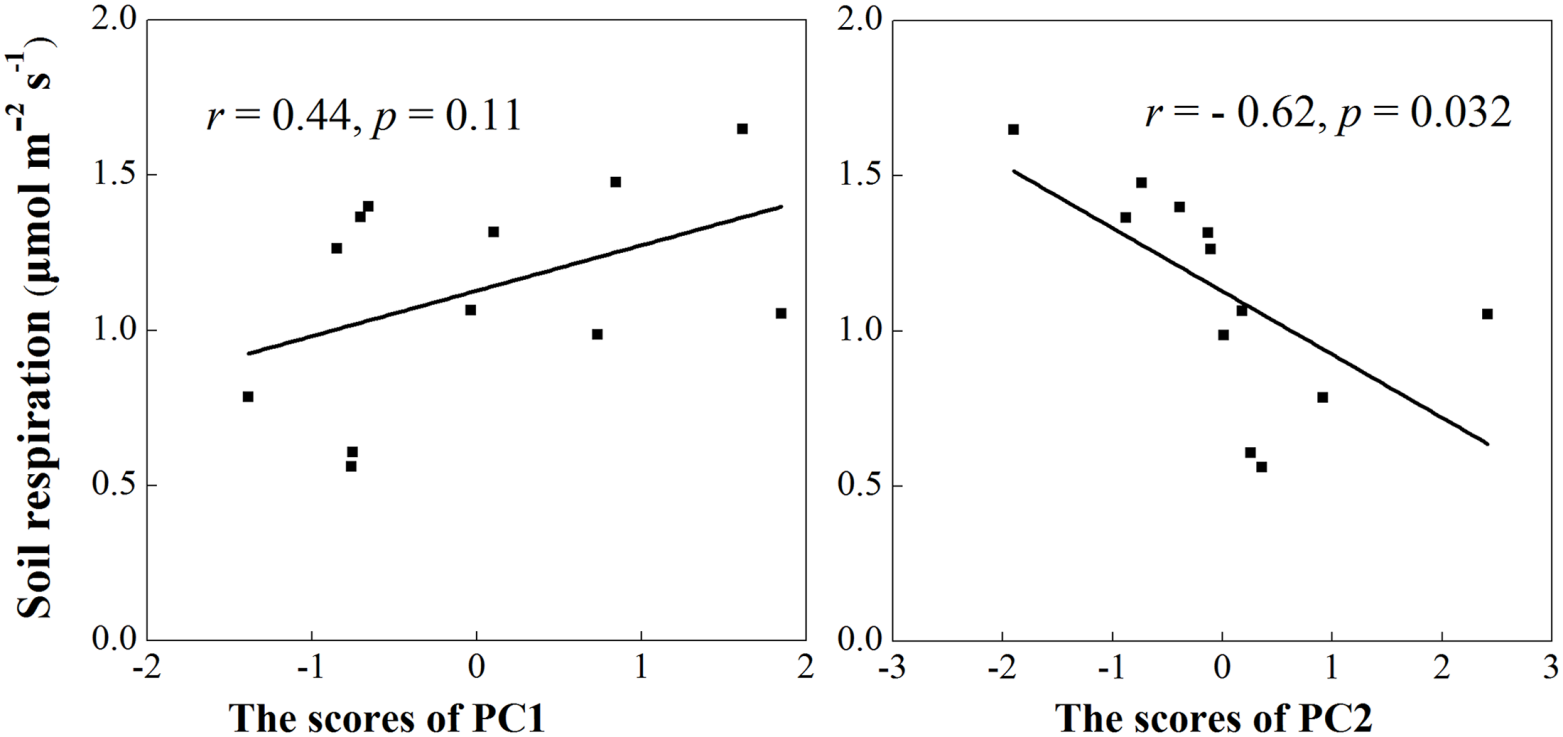
Relationships between the PCA scores of PLFAs and annual soil respiration (*n* = 12). Linear regression lines with corresponding *r*-values and levels of statistical significance are shown.

The results showed that R_s_ had a highly positive correlation with soil temperature but a negative correlation with soil moisture (Fig 6). Moreover, the PC2 score was significantly negatively correlated with the annual R_s_ (Fig 7).

## Discussion

### Soil respiration

Girdling decreased R_s_ by 40.5% during the experimental periods. This result suggested that the allocation of photosynthates to the root system controls R_s_ [3, 4, 45], because girdling terminated the allocation of photosynthates from the tree crown to the roots. Depletion of carbohydrate in fine roots decreased R_A_ which was mainly responsible for the reduction of R_s_ [45, 46]. Although, the dynamics of non-structure carbohydrate content in fine roots was not investigated, the significantly increased soil NO_3_^−^-N concentration can support the speculation (Table 2). Previous studies also indicated that the increased NO_3_^−^-N concentration was mainly attributed to the termination of plant N uptake [47–49] because of the reduction in mycorrhizal hyphae and fine roots after girdling [50]. Additionally, soil microbes are generally limited by available C, and the decrease in labile C inputs by rhizodeposition after girdling may inhibit microbial respiration, which contributed to the decrease in R_s_. Moreover, the reduction of soil microbial biomass and the alteration of microbial community composition after girdling (Table 3) should be partly responsible for the reduction in R_s_. Treseder [15] also found that soil microbial biomass reduced with decreasing sol microbial respiration in different ecosystems.

However, no decrease in R_s_ was observed in the first three months after girdling, which was consistent with some previous studies [4, 51]. Chen et al. [4] considered that no decrease in R_s_ in the first two months in *Eucalyptus urophylla* plantations was mainly due to the resprouting ability of *E*. *urophylla*, which had some living roots and released CO_2_ after short-time girdling [13, 45]. Edwards and Ross-Todd [51] also found little reduction in R_s_ in the girdling plots with tulip poplar species, which had resprouting ability. In this study, *C*. *lanceolata* can also resprout, and some roots may be alive and continue to release CO_2_ after girdling. The resprouting ability relies on the starch reserves in the roots [13]. Unfortunately, in the present study, further exploration of the inherent mechanism is hampered by the limited data on starch reserves in the roots. Additionally, decomposition of dead fine root and associated mycorrhizal hyphae may result in a short-term increase in the contribution of R_H_ to R_s_ after girdling which can offset the decreased R_A_ [45, 52].

Our results showed that tree girdling significantly decreased R_s_ which indicated girdling was a powerful method to distinguish R_A_ and R_H_. However, there may be some limitations when using girdling to distinguish R_A_ and R_H_ [13]. For example, the roots may be alive in short time after girdling which results in underestimation of R_A_ and overestimation of R_H_. In addition, girdling may decrease transpiration and alter soil water content which can also affect R_H_ [26].

No significant difference in Rs was observed between CT and N plots (Fig 3), which suggested that N addition did not affect Rs. This result was contrary to our hypothesis that N addition would decrease R_s_ and was also inconsistent with some previous studies [15, 16, 18, 22]. The unchanged Rs under N addition in the studied subtropical forest may be explained by the following reasons. Response of Rs to N addition was dependent on the rate of N addition. In this experiment, the total amount of N added to the soil was 100 kg N ha^−1^. Mo et al. [17] also observed that N addition of 100 kg N ha^−1^ yr^−1^ in a tropical forest did not change R_s_, but N addition of 150 kg N ha^−1^ yr^−1^ significantly decreased Rs. Additionally, these conflicting results may be attributed to the different responses of R_H_ and R_A_ to N addition [10]. In the present study, R_H_ and R_A_ had different responses to N addition, i.e., reduced R_H_ but increased R_A_ (Fig 5). Numerous previous studies also found the reduction in R_H_ after N addition [30, 53–54]. This reduction may be attributed to the suppression of the soil organic matter decomposition under high N availability. Moreover, the decrease in R_H_ can also be explained by the decreased soil microbial biomass measured by PLFA analysis after N addition because soil microbes play an important role in mineralizing soil organic matter [55, 56]. The increase in R_A_ under N addition (Fig 5) was in agreement with the observations of Jia et al. [57] which reported that the average RA rates in *Larix gmelinii* and *Fraxinus mandshurica* plantations increased by 10% and 13% respectively in fertilized plots. The increase in R_A_ was likely due to increases in fine root biomass and rhizosphere microorganisms after N addition or fertilization [22, 57, 58].

N addition augmented the negative effect of girdling on R_s_, which was consistent with our hypothesis. This finding indicated that the effect of C availability on R_s_ was dependent on soil N availability. Janssens et al. [23] also found decreased R_s_ under higher N availability which may have been caused by the suppression of decomposition of soil organic matter by N addition. In the same study site, Wang et al. [18] also found that the increase in N availability was beneficial to the reduction of soil organic matter mineralization and increased soil C sequestration to a certain extent. The lower soil microbial biomass in the GN plots than in the N plots and the difference in the soil microbial community between N and GN plots (Fig 2) were observed, and a negative correlation existed between the annual R_s_ rate and PC2 score (Fig 7). Therefore, the decrease in the soil microbial biomass and the shift in the soil microbial community composition after N addition were mainly responsible for the augmentation of the negative effect of girdling on R_s_ [15, 23].

### Soil microbial community composition

The decrease in bacterial and Gram-negative bacterial PLFAs in the G plots suggested that girdling altered soil microbial community, which was consistent with our hypothesis and several previous findings [47, 59, 60]. The decrease in bacterial PLFAs after girdling was most likely due to the depletion of labile C in the soil. The C availability to soil microorganisms is ultimately derived from plant photosynthesis, thus the processes or factors which alters the photosynthesis C allocation to soil can affect microbial biomass and activities. Soil labile C availability has been assumed to be the most common limiting factor to soil bacterial growth [34]. The decrease in soil microbial biomass after girdling suggested that soil microorganisms were C-limited in subtropical forest ecosystem. The decrease in Gram-negative bacteria induced by girdling confirmed speculation because Gram-negative bacteria are generally favored by labile C substrates [44, 61].

N addition significantly decreased bacterial biomass and altered the soil microbial community composition, which agreed with the results of previous studies [62–65]. However, N addition did not change fungal biomass, which was consistent with previous studies [15, 64]. This finding suggested that bacterial biomass was more negatively affected by N addition than fungal biomass. The greater decrease in the concentration of Gram-negative bacteria than in the Gram-positive bacteria after N addition resulted in a higher ratio of Gram-positive to Gram-negative bacteria in N addition plots. Similar results were also observed in previous studies [18, 66], suggesting that N addition inhibited the growth of Gram-negative bacteria more effectively than the growth of Gram-positive bacteria. Additionally, the timing of N addition was also a key factor controlling the effect of N addition on microbes [15, 67, 68]. The increase in N availability may increase microbial biomass and shift community composition to N-lovers shortly after N addition. Bai et al. [67] also found a transient increase in soil N_2_O emission which was controlled by nitrifier and denitrifier in response to N addition in a temperate forest on Mt Changbai, and the increased emission only lasted for two weeks. However, Sun et al. [69] found unchanged microbial biomass in the same site under N addition which may be caused by the confounded response of different microbial communities to N addition. Wan et al. [68] found that N additions for eight months did not change the biomass and structure of the soil microbial community under *Mytilaria laosensis* and *C*. *lanceolata* which may be due to the short duration of their N addition experiment. A meta-analysis carried out by Treseder [15] suggested that reductions in the abundance of microbes and fungi were more evident in studies with longer durations and higher total amounts of N application.

Similar to our hypothesis, PCA results showed that N addition altered the effect of girdling on the composition of the soil microbial community, indicating that N availability changed the effects of C supply on the microbial community structure. Demoling et al. [65] demonstrated that the C limitation of soil bacteria became more evident in N-fertilized plots, especially in previously N-limited forests. The C limitation of soil bacteria in the G plots was likely increased by N addition, which resulted in the alteration of soil microbial community composition.

In conclusion, several important findings were noted in this study. First, N addition augmented the negative effect of girdling on R_s_, suggesting that an increase in soil N availability can modify the response of R_s_ to C allocation. In the context of climate change, N deposition will decrease CO_2_ emission from the decomposition of soil organic matter and benefit soil C sequestration in subtropical forest ecosystems, particularly when soil C availability is decreased. Second, N addition altered the effect of girdling on soil microbial community composition, suggesting that reductions in belowground C allocation by plants have a greater effect on soil microbial community composition under the N deposition than under ambient condition.
